# A Flow Adhesion Assay to Study Leucocyte Recruitment to Human Hepatic Sinusoidal Endothelium Under Conditions of Shear Stress

**DOI:** 10.3791/51330

**Published:** 2014-03-21

**Authors:** Shishir Shetty, Christopher J. Weston, David H. Adams, Patricia F. Lalor

**Affiliations:** ^1^NIHR Biomedical Research Unit, Centre for Liver Research, School of Immunity and Infection, University of Birmingham

**Keywords:** Immunology, Issue 85, Leucocyte trafficking, liver, hepatic sinusoidal endothelial cells, peripheral blood lymphocytes, flow adhesion assay

## Abstract

Leucocyte infiltration into human liver tissue is a common process in all adult inflammatory liver diseases. Chronic infiltration can drive the development of fibrosis and progression to cirrhosis. Understanding the molecular mechanisms that mediate leucocyte recruitment to the liver could identify important therapeutic targets for liver disease. The key interaction during leucocyte recruitment is that of inflammatory cells with endothelium under conditions of shear stress. Recruitment to the liver occurs within the low shear channels of the hepatic sinusoids which are lined by hepatic sinusoidal endothelial cells (HSEC). The conditions within the hepatic sinusoids can be recapitulated by perfusing leucocytes through channels lined by human HSEC monolayers at specific flow rates. In these conditions leucocytes undergo a brief tethering step followed by activation and firm adhesion, followed by a crawling step and subsequent transmigration across the endothelial layer. Using phase contrast microscopy, each step of this 'adhesion cascade' can be visualized and recorded followed by offline analysis. Endothelial cells or leucocytes can be pretreated with inhibitors to determine the role of specific molecules during this process.

**Figure:**
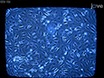


## Introduction

It is now well established that leucocyte recruitment in general follows the paradigm of the multistep adhesion cascade^1^. This involves the capture of leucocytes from flowing blood by endothelial cells lining the vessel wall. Initially, leucocytes undergo a rolling step which is mediated by selectin receptors or members of the immunoglobulin superfamily. This allows G-protein coupled receptors (GPCRs) expressed on the leucocyte surface to be activated by chemokines presented on the endothelial glycocalyx. This leads to the alteration of integrin confirmation to a 'high affinity' state on the leucocyte surface and arrest and firm adhesion to the endothelium. Firm adhesion is then followed by shape change and crawling of the leucocyte on the vessel. The final step is transmigration through the endothelial monolayer, which can occur via paracellular or transcellular routes.

While the multistep adhesion cascade describes the general mechanism of leucocyte recruitment within the body there are organ specific differences. In the liver the majority of leucocyte recruitment occurs within the hepatic sinusoids in contrast to other organs where recruitment generally occurs within the post-capillary venules^2^. The hepatic sinusoids are a low shear environment and leucocytes undergo a brief tethering step prior to firm adhesion which is selectin independent^2^. These channels are lined by the hepatic sinusoidal endothelium which is discontinuous and contains fenestrae, open pores 100-200 nm in diameter, and lack a basement membrane^3^. Elucidating the molecular mechanisms that mediate leucocyte recruitment across human hepatic sinusoidal endothelium could identify organ specific therapeutic targets for inflammatory liver diseases.

Flow adhesion assays are essential tools in studying leucocyte recruitment. They allow the reconstruction of leucocyte recruitment in presence of shear stress to analyze adhesion under well-defined forces. The most frequent use for the assay is the study leucocyte adhesion to cultured endothelial monolayers or purified substrates. Commercially available flow chambers are used to perfuse cells under conditions of laminar flow between two flat surfaces and then visualize the dynamic process of adhesion on a microscope^4^. Previous groups have demonstrated that certain adhesive interactions only take place under flow and cannot be studied in static assays^5,6^.

We have used this technique to recapitulate the hepatic sinusoids and study leucocyte recruitment under conditions of low shear stress. Primary human HSEC are cultured in microslides and leucocytes can then be perfused over this monolayer at a rate of flow calculated to reproduce the shear stress within hepatic sinusoids. The shear stress is a stress that is applied parallel or tangential to a surface as opposed to normal stress which is perpendicular. Any fluid that is moving along a boundary will exert a shear stress on that boundary. Shear stress has been shown to be an essential component of lymphocyte transmigration^7^. Under these conditions each step of the adhesion cascade can be visualized by phase contrast microscopy. This method has allowed important insights into the recruitment of leucocytes within the liver including the study of conventional adhesion molecules^8^, the role of chemokines and chemokine receptors^9-11^, and atypical adhesion molecules such as the vascular adhesion protein-1 (VAP-1)^8,12 ^and common lymphatic and vascular endothelial receptor-1 (CLEVER-1)^13^. Whilst this assay has been mentioned in several of our group's publications, its description has been brief and we have taken this opportunity to provide a detailed step by step guide to help in troubleshooting and prevent technical errors when attempting the assay. Furthermore, we have recently changed the sourcing of microslide chambers which allows accurate alterations in shear stress. We believe this broadens the applicability of the assay to other endothelial and immune cells. The following method describes the preparation and technique for carrying out a flow based adhesion assay with human hepatic sinusoidal endothelial cells and peripheral blood lymphocytes.

## Protocol

### 1. Microslide Preparation

1. Precoat a six channel microslide with rat tail collagen type I (RTC, diluted in PBS 1:100 giving a working dilution of 220 µg/ml). This is performed by injecting 30 µl of diluted RTC solution directly into the channels and incubating for 2 hr at 37 °C, followed by three washes with PBS.

### 2. Seeding Cells in Microslides

1. Dissociate a confluent T75 flask of cultured human hepatic sinusoidal endothelial cells (HSEC) (isolated from liver tissue as described previously^13^) in trypsin-EDTA, wash in PBS, and resuspend at 3 x 10^6^ cells/ml in complete media (human endothelial basal media supplemented with 2 mM L-glutamine, 100 U/ml penicillin and 100µg/ml streptomycin, 10% heat-inactivated AB human serum, 10 ng/ml of vascular endothelial growth factor and 10 ng/ml of hepatocyte growth factor). Inject 30 µl of cell suspension directly into each channel.
2. Leave cells to adhere for 1 hr in a humidified incubator at 37°C with a 5% CO_2_ atmosphere on a slide rack. After allowing the cells to adhere fill the ports on either side of each channel with complete medium (**Figure 1**).

### 3. Cytokine Stimulation of Cells

1. Leave cells in incubator for 24 hr. Assess cell growth using an inverted phase contrast microscope. 24 hr prior to adhesion assay, stimulate the endothelial monolayers with cytokine by replacing the growth media with complete media supplemented by tumor necrosis factor alpha and interferon gamma, both at 10 ng/ml.

### 4. Isolation of Peripheral Blood Lymphocytes

1. Isolate peripheral blood lymphocytes from whole blood by initially purifying the mononuclear fraction by density gradient centrifugation over an appropriate cell separation centrifugation media for 25 min at 800 x g. Incubate the cells on plastic for 1 hr to allow adherence of monocytes.
2. Following adherence to plastic aspirate the lymphocyte enriched supernatant. Wash the lymphocytes and resuspend them (typically 1 x 10^6^ cells/ml in flow medium (endothelial basal media containing 0.1% (v/v) bovine serum albumin (BSA)).

### 5. Pretreatment of Endothelium or Leucocytes with Inhibitors

1. Prepare recommended dilutions of function blocking antibodies or small molecule inhibitors in endothelial media/0.1% (v/v) BSA. Replace complete medium with blocking antibody/inhibitor solution in chosen microslide channel 30 min prior to flow assay.
2. Where pretreatment of chemokine receptors on lymphocytes are planned, resuspend isolated leucocytes in RPMI solution containing 0.1% (v/v) BSA and incubate with 200 ng/ml of Pertussis Toxin to block G-protein coupled receptor activity of chemokine receptors; alternatively dilute specific function blocking antibodies or small molecule inhibitors of chemokine receptors at recommended dilution. Incubate leucocytes at 37 °C for 30 min, then wash and resuspend in flow medium.

### 6. Setup of Flow Assay System

1. Prewarm a thermostatically controlled transparent chamber to 37°C. The chamber should have ports for the insertion of silicone tubing and electronic supply for an electronic solenoid valve that enables switching between cells and media with virtually no dead volume. The chamber is mounted on an inverted microscope to allow phase contrast microscopy. **Figure 2** demonstrates the flow assay chamber and microscope and microslide placed on the microscope stage.
2. Prefill a 50 ml glass syringe with a luer lock with 10 ml of sterile distilled water and attach a length of 25 cm silicone tubing to the syringe port. Insert in a syringe pump. Alter the withdrawal rate of the syringe pump according to the microslide manufacturer's instructions to maintain a shear stress of 0.05 Pa (0.5 dyne/cm^2^, **Figure 3**).
3. Take two 5 ml syringes, discard the plungers and attach the barrels to the two in-flow ports of an electronic solenoid valve using silicone tubing.
  1. Attach 12 cm of silicone tubing to the out-flow valve. The valve permits alternation between a cell free wash buffer (endothelial media/0.1% (v/v) BSA) and the lymphocyte suspension.
  2. Flush the electronic solenoid valve by inserting wash buffer in both syringe barrels and ensuring buffer is flowing from one barrel through the valve and into the out-flow silicone tubing.
  3. Use the valve switch to alternate to the other barrel and ensure the buffer is flowing and that all bubbles are removed from the system. Remove wash buffer from one of the barrels and replace with lymphocyte suspension **Figure 4a.**
4. Connect the silicone tubing from the syringe pump to one port of a chosen microslide channel via a microslide adaptor. Then connect the silicone tubing from the outflow valve to the opposite port via a microslide adaptor. Ensure that the silicone tubing and the adaptors are filled with wash buffer prior to connection to prevent air bubbles entering the system (**Figure 4b**).
5. Once attached to the flow system, place the microslide on the microscope stage and attach with clips or tape to prevent movement. Set the microscope to the 10X objective together with the appropriate phase setting. Ensure the endothelial monolayer is visualized and in focus using the ocular lens. Ensure images can be captured via a camera which is attached to the microscope whereby images can be relayed to a monitor and recorded.

### 7. Flow Assay Technique and Recording of Adhesion for Analysis

1. Perfuse the endothelial layer with wash buffer for 2 min by commencing withdrawal of the syringe pump to remove any debris or unbound blocking antibody and then switch the valve to allow a 5 min bolus of leucocyte solution at a constant wall shear stress of 0.05 Pa.
2. During the last 2 min of the leucocyte bolus, record 10 random fields along the length of the microslide. This allows offline analysis of lymphocyte rolling/tethering on the endothelial monolayer. Record each field for approximately 10 sec before moving to the next and ensure that recordings are made against the direction of flow to avoid recording the same rolling cell twice in quick succession.
3. Follow the leucocyte bolus with a 5 min bolus of wash buffer by returning the valve to its start position. During the final 2 min of this bolus carry out a second recording phase by choosing at least 10 randomly selected fields along the length of the microslide. Record each field for approximately 5 sec before moving to the next and ensure that the endothelial monolayer and adherent leucocytes are in clear focus. This allows offline analysis of the pattern of adhesion.

## Representative Results

This assay has the ability to visualize the multistep flow adhesion cascade and elucidate the underlying molecular mechanisms by comparing results of control experiments to those with molecular inhibitors. Various vascular beds can be recapitulated by incorporating specific endothelial cells and altering shear stress conditions.

Each step of the adhesion cascade can be analyzed offline by following the recording method outlined in the protocol. The first step of the adhesion cascade is the rolling of leucocytes which can be expressed as a percentage of total adherent cells. Offline analysis allows the number of adherent cells to be enumerated in each recorded field during the leucocyte bolus. Playback of the image allows the comparison of cells that are firmly adherent and those that are undergoing a rolling motion across the endothelium. Rolling motion can be visualised using this technique, each field is recorded for at least 10 sec. Rolling cells are identified by their reduced velocity over the endothelial surface compared to flowing cells. This behaviour must be demonstrated for at least 5 sec without detachment. The adhesion cascade within the hepatic sinusoids takes place in a low shear environment and *in vivo* studies have confirmed minimal rolling with only a brief tethering step. We have confirmed that the flow assay reflects the environment of the hepatic sinusoids by demonstrating that fewer than 10% of adherent leucocytes persistently roll over stimulated HSEC in these assays.

The next step of the adhesion cascade is firm adhesion. Total adherence can be calculated from the second stage of recording during the wash buffer bolus (step 7.3). Offline analysis allows the total number of firmly adherent cells to be counted in each field (**Figure 5**). Firmly adherent cells are defined as cells that are stationary or shapechanged with slow crawling behavior. The average number of cells per field can then be calculated. This figure can then be used, in conjunction with the total surface area of the field of view (determined using a graticule or equivalent), concentration of lymphocytes (typically 1 x 10^6^ cells/ml) and the flow rate to express the extent of lymphocyte adherence as adherent cells/mm^2^/10^6^ cells perfused.

Studying the pattern of adhesion involves the analysis of the last two steps of the adhesion cascade including shape-change, crawling and transendothelial migration. Leucocytes adherent to the upper surface of the HSEC monolayer appear phase-bright whilst those that have migrated through the monolayer appear phase dark (**Figure 6**). The cells can then be classified as exhibiting 'static' adhesion (nonmigrated/ round), 'shape-changed' morphology or as 'migrated' and individual categories are then expressed as a percentage of the total adhesive population.


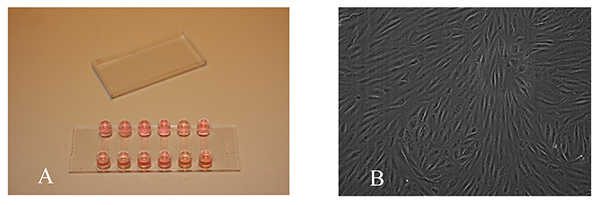
**Figure 1. Monolayer of primary human hepatic sinusoidal endothelial cells within flow chamber. A**) Microslides filled with media containing monolayer of endothelial cells prior to commencement of flow adhesion assay. **B**) Phase contrast image of confluent endothelial monolayer, endothelial cells should be seeded in microslide which have been precoated (for human hepatic endothelial cells this should be with rat tail collagen type 1) and it is essential that the endothelial cells are healthy in culture and confluent. Click here to view larger image.


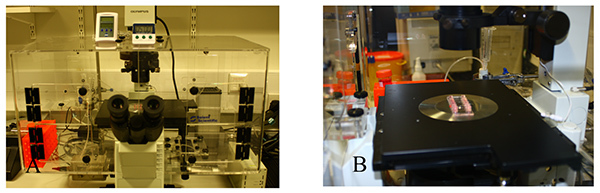
**Figure 2. Flow assay chamber.** A flow assay chamber set-up can be seen here, it consists of a transparent chamber which is mounted on an inverted microscope. A heater is placed in the chamber and should be thermostatically controlled to maintain a temperature of 37 °C. There should be ports available to connect silicone tubing from a microslide within the chamber to a syringe pump which is located outside. The microslide is placed directly on the microscope stage. Click here to view larger image.


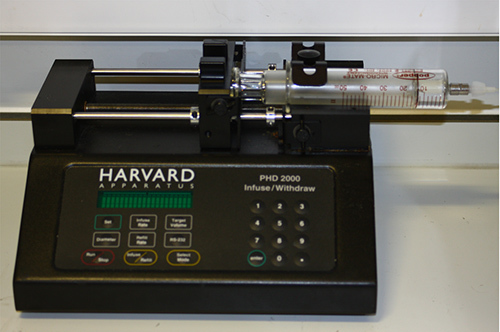
**Figure 3. Syringe Pump**. A syringe pump is connected via silicone tubing to the flow chamber. The pump is set to a specific withdrawal rate depending on the desired shear stress required for the assay. Click here to view larger image.


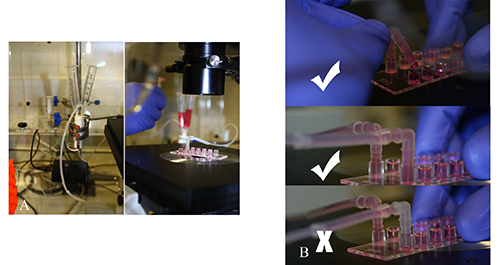
**Figure 4. Connecting valve to flow chamber.A**) An electronic solenoid valve allows switching between two syringe barrels containing either cells or media with virtually no dead space. **B**) Once the valve is flushed and the two barrels are set up, the silicone tubing from the valve is connected to the flow chamber. It is critical that when connecting the adaptor on the silicone tubing to the port on the flow chamber there is a liquid/liquid interface. Click here to view larger image.


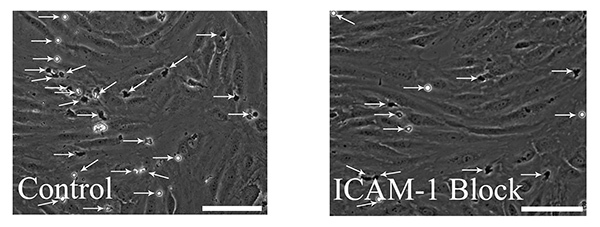
**Figure 5. Measurement of total leucocyte adherence.** During the last two minutes of the wash buffer bolus step (as outlined in the protocol), a minimum of ten random fields should be recorded. These can be analyzed off-line and the total number of firmly adherent cells can be counted in each field. Total adhesion of leucocytes can be compared between control chambers and those pretreated with blocking antibodies, here we show a representative field from a control slide and a slide pretreated with intracellular adhesion molecule-1 (ICAM-1) blocking antibody. Arrows have been added to highlight the adherent leukocytes, in the representative field from the control slide there are a total of 25 leukocytes identified and in the ICAM-1 block slide there are a total of 13 leukocytes identified. Scale bars = 100 µm. Click here to view larger image.


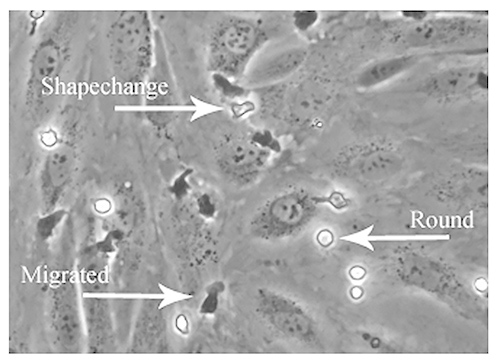
**Figure 6. Analysis of the pattern of leucocyte adhesion on endothelial monolayers by phase contrast microscopy.** Offline analysis of recorded fields can also be used to study the direction and velocity of leucocyte adhesion. Specific steps of the adhesion cascade can be visualized and quantified using phase contrast imaging. Phase bright cells which are firmly adherent but not activated can be termed 'round' adhesion, the cells which are activated and phase bright can be termed 'shape changed' and the cells which are phase dark are the cells which have undergone transendothelial migration and can be termed 'migrated'. The image shows examples of each pattern of adhesion. Click here to view larger image.

## Discussion

The most critical step for successfully performing a flow assay is ensuring that a healthy and confluent monolayer of endothelial cells is ready prior to the flow adhesion assay. Primary endothelial cells can be difficult to culture and sensitive to alterations in culturing methods. It is important that 1) flow chambers are adequately and uniformly coated with endothelial cells in a monolayer; for HSEC we use rat tail collagen type I but this may differ for other endothelial populations, 2) culture medium is appropriate for the cell type, for HSEC we have described our complete medium in the protocol section. Other vital steps include setting the syringe pump at the appropriate rate to reflect physiological levels of shear stress.

During the flow assay it is necessary to prevent air bubbles within the flow circuit which can damage the endothelial monolayer or strip immune cells from the endothelial surface. This can be prevented by ensuring that all silicone tubing and adaptors are perfused with wash buffer prior to connection, that all air bubbles are removed and that the media are prewarmed prior to use. When connecting the adaptors to the ports on the microslide it is very important that there is a liquid/liquid interface during connection, if there is any air then this will form an air gap within the system which will disrupt the endothelial monolayer during the syringe withdrawal step. The leucocyte solution in the syringe barrel needs regular agitation to ensure that the cells do not settle, thus maintaining a constant cell density throughout the experiment.

During the recording steps it is important to ensure the image of the endothelial layer is adequately focused and clear to allow accurate offline analysis, and that during the second step of the flow assay (post leucocyte bolus) that enough time is left during the wash buffer phase before recording is recommenced to ensure that all nonadherent leucocytes are removed. Similarly it is essential to use endothelial cells in a monolayer of appropriate density to prevent loss of cells which can interfere with flow patterns in narrow capillaries and also can be hard to discriminate from larger adherent leucocytes under phase contrast microscopy. We have described optimal seeding density for human hepatic sinusoidal endothelial cells but this may vary between different endothelial populations and species.

Significant progress has been made in studying leucocyte recruitment in animal models with intravital microscopy. The major advantage of the flow adhesion assay method is that leucocyte recruitment can be studied in a binary system with primary human endothelial cells. Furthermore, these interactions can be studied under physiological relevant levels of shear stress. It is important to confirm findings of intravital studies in animals with human cellular systems as there may be differences in endothelial properties between species. One of the limitations of the flow assay is that leucocyte recruitment is being studied in a unicellular environment of the endothelial monolayer. In addition once the leucocytes have adhered and transmigrated across the endothelium they may not be in present in sufficient numbers to be isolated and subjected to downstream processes.

Despite these limitations, once the flow adhesion assay has been mastered it can be developed to perform further analysis of the leucocyte adhesion cascade and adapted to recapitulate a multicellular environment. Prolonged recording of single fields and the use of tracking software can be used to analyze crawling behavior of the leucocytes. Furthermore upon completion of the flow assay the microslides can be interrogated using laser scanning confocal microscopy and immunofluorescent labeling to study adhesion and transmigration in more detail. Additionally, we have previously developed an* in vitro* model where flowing leucocytes could interact with hepatic endothelium conditioned by the presence of hepatocytes. This assay can also be developed to study subpopulations of leucocytes: our group has performed studies with subsets such as regulatory T cells, B cells, and liver infiltrating leucocytes.

These studies are evidence that the flow adhesion assay is a powerful tool to study general and organ specific leucocyte recruitment in human systems.

## Disclosures

The authors declare no competing financial interests
